# Dengue fever in a south Asian metropolis: a report on 219 cases

**Published:** 2017-06

**Authors:** Shiv Sekhar Chatterjee, Ankush Sharma, Shilpee Choudhury, Sushil Kumar Chumber, Ras Bage, Nittin Parkhe, Uma Khanduri

**Affiliations:** 1 Department of Laboratory Diagnostic Services, St Stephen Hospital, Delhi, India; 2 Department of Medicine, St Stephen Hospital, Delhi, India; 3 Department of Radiology, St Stephen Hospital, Delhi, India

**Keywords:** Dengue fever, Flavivirus, Dengue Hemorrhagic fever, Dengue shock syndrome

## Abstract

**Background and Objectives:**

Yearly epidemics of Dengue fever occur post-monsoon in India’s capital, Delhi. A prospective observational study was conducted during the outbreak months to understand the epidemiology and outcome of this infection and its economic impact.

**Materials and Methods:**

Febrile hospitalized (n=219) patients with dengue fever diagnosed by a combination of MAC-ELISA, GAC-ELISA and NS1Antigen-ELISA were enrolled. Epidemiologic (including economic) parameters, clinical, radiological and laboratory manifestations were noted and patients followed up over the period of hospital stay. Patient management means and outcome were recorded and analysed.

**Results:**

As per WHO-2009, 153 (69.9%) and 27 (12.3%) patients were classified as dengue with warning signs and Severe Dengue respectively while according to WHO-1997 guidelines 39 (17.8%) and 18 (8.2%) patients were classified as DHF and DSS respectively. 216 patients were from the city while three were travellers; hospitalization was more frequent among the young and male gender. Fever, vomiting, aches and abdominal pain were the most common troublesome manifestations; classical dengue triad was present in 55 (25.1%) patients; hemorrhagic, neurologic and mucocutaneous manifestations were present in 44 (20.1%), 8 (3.7%) and 70 (32%) patients. Ascitis, pleural effusion, and Gall bladder wall oedema was found in 53 (24.2%), 31 (14.1%) and 45 (20.5%) patients respectively. Mortality was 1.4% (3 deaths); in addition there was an intra-uterine fetal death; mean expenditure per patient during the illness was US$ 377.25.

**Conclusion:**

Dengue virus infection results in immense morbidity and substantial mortality.

## INTRODUCTION

Dengue, a mosquito borne Flavivirus infection, leads to recurrent epidemics in urban agglomerates causing immense morbidity and occasional mortality (1). Though world-wide in distribution, dengue poses a bigger challenge in resource poor countries, like India, especially for the urban and rural poor. Recently, World Health Organization (WHO) has modified guidelines for management of dengue infection (2). Previous classification of Dengue Hemorrhagic fever (DHF) and Dengue shock syndrome (DSS) have been replaced by the new triage of Dengue without warning signs, Dengue with warning signs and Severe Dengue to expedite adequate therapy (2). Criteria for presumptive diagnosis, hospital admission, fluid and blood component administration, and discharge have been forwarded. These may significantly impact dengue mortality; however their usefulness is not validated in India (2). Repeated dengue epidemics occur in Delhi, National Capital Region, India (1, 3–5) where a large proportion of people live under the poverty line (6) or in unhygienic conditions (6). We planned this study to highlight the clinical and laboratory features of acute dengue infection and its financial impact on the affected patients.

## MATERIALS AND METHODS

A prospective observational study was undertaken at St Stephen Hospital, Delhi from August 2013 to January 2014. A total of 918 suspected dengue patients were tested with for NS1 antigen, anti-dengue IgM and IgG antibodies by ELISA. Antibody testing was done with commercially available Panbio Dengue IgM capture ELISA (Inverness Medical Innovations, Cat No. E-DEN01M/EDEN01M05) (MAC-ELISA) and Dengue IgG capture ELISA (Inverness Medical Innovations, Cat No. E-DEN02G) (GAC-ELISA). One Negative control, one Reactive Control and Callibrators in triplicate was used in each ELISA test as per manufacturer instructions. Index value [ratio of sample absorbance and cut-off value (calculated from calibrator ODs and calibration factor given in each kit)] and Panbio units were calculated for each sample and results are classified as positive [Index value >2.2], negative (Index Value<1.8) and equivocal (Index Value∼1.8–2.2) according to the manufacturer’s instructions. Any initial equivocal result was retested to confirm the result. NS1 antigen (NS1Ag) testing was done with commercially available Platelia^TM^ Dengue capture 96-well sandwich format ELISA (Bio-Rad, Cat. No.72830). One Negative control, two calibrators, and one Positive Control were used in each run, and the cut-off was calculated as mean of calibrators. All ELISAs were performed on a fully automated EVOLIS platform.

### Inclusion criteria for observational study.

Febrile hospitalized patients with either i) MAC-ELISA and GAC-ELISA reactive, or ii) MAC-ELISA alone reactive, or iii) NS1Ag-ELISA and MAC-ELISA reactive or iv) NS1Ag and GAC-ELISA were included in the study (n=219).

### Exclusion criteria for observational study.

1) Patient tested with rapid Immunochromatographic test for Dengue antibodies or Antigen, 2) Patient treated on outpatient basis (not admitted), 3) Patient MAC-ELISA and NS1Ag-ELISA negative, GAC-ELISA alone reactive, 4) Febrile patient with blood culture positive bacterial sepsis or significant pyuria and bacteriuria or malaria parasite documented by thick, thin films and antigen detection test.

Informed consent was taken from each patient enrolled in the study. Institutional ethics committee approval was taken for conducting this study. Patient’s occupation, daily income, loss of income if any, total expenses due to the illness were recorded. Meticulous clinical examination was carried out daily; all patients underwent the following investigations as per the clinical requirement–complete blood count (CBC), blood culture, urine culture, thin and thick smears for malarial parasite, rapid malarial antigen testing (Sure Test, pan-LDH-2 antigen detection, Microgene Diagnostics), Erythrocyte Sedimentation Rate (Westergren’s method)), liver function tests (LFT), renal function tests, chest X-ray (CXR) and ultrasonography (USG). CBC was done daily for the first 4 days of hospital stay and then as and when required depending on the clinical situation. Clinically, presence of tachypnea, chest retractions, decreased breath sounds and decreased vocal resonance were considered signs of pleural effusion. Presence of abdominal distension with fullness of the flanks and presence of shifting dullness or fluid thrill was taken as evidence of ascites. The extent of hemoconcentration was quantitated by taking a difference between the maximum hematocrit at admission or anytime during the hospital stay and the minimum hematocrit recording at convalescence or discharge (7). Dengue with warning signs was defined as laboratory confirmed dengue with any of the following: abdominal pain or tenderness, Persistent vomiting, clinical fluid accumulation, mucosal bleed, lethargy, restlessness, Liver enlargement >2 cm and increase in hematocrit (>20%) concurrent with rapid decrease in platelet count (to below 40000/ul) (2). Severe dengue was defined as patients with any of the following features: severe plasma leakage with shock and/or fluid accumulation with respiratory distress, severe bleeding, or severe liver, renal, cardiac, and pulmonary or central nervous system impairment (2). DHF was diagnosed as per the older WHO guidelines as a probable case of dengue fever with hemorrhagic tendencies and thrombocytopenia along with the presence of evidence of plasma leakage manifested by any one or more of the following i.e., a rise in the average hematocrit for the age and sex by >20%; a >20% drop in the hematocrit following volume replacement compared to the baseline; signs of plasma leakage i.e., pleural effusion, ascites, hypoproteinemia (7). The area specific hematocrit cut off values for hemoconcentration was defined as >36.3% in less than 12 years age group (4) and >37.5% in those more than 12 years (8). Criteria for splenomegaly on USG was span more than 11 cm on greatest dimension or weight greater than 250gm (9) and that for hepatomegaly was span at mid-clavicular line greater than 15.5 cm (10). Guidelines of the National Vector Borne Disease Control Program (NVBDCP) in India were used as criteria for assessing appropriateness of platelet transfusion (11). Each patient’s total expenditure was determined from the final bill generated for each patient. Statistical analysis was carried out on the data in Microsoft Excel software.

## RESULTS

**Fig. 1:**
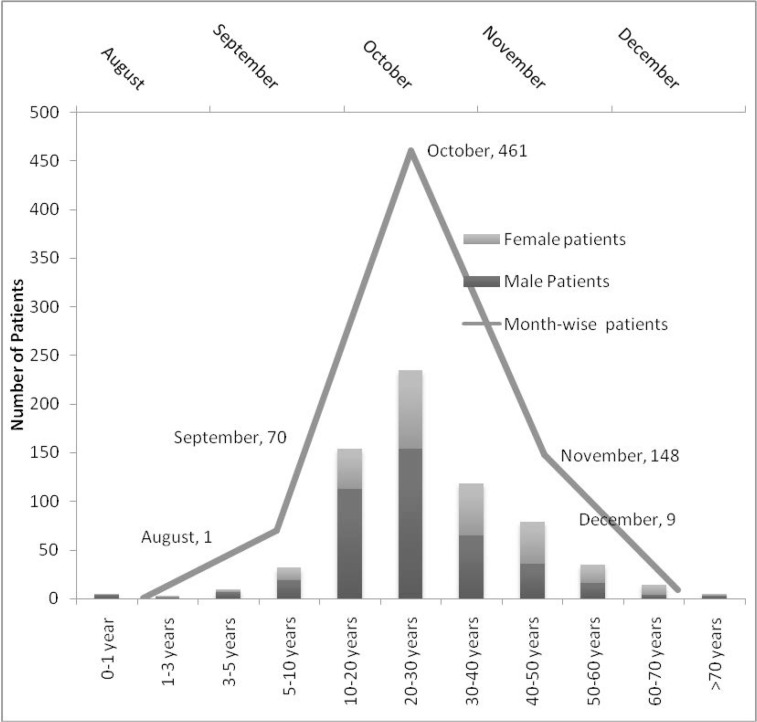
Age, gender distribution and month of presentation of Dengue fever patients

During 2013, out of 918 suspected dengue patients (572 males, 346 females), 689 [422 (61.2%) males, 267 (38.8%) females) were reactive in any of the three tests, MAC-ELISA, GAC-ELISA or NS1Ag-ELISA (Table 1). The various combinations of positive tests noted in these patients are shown in Table 1. Maximum and minimum incidence of dengue was in the age group of 20–30 years (235, 34.1%) and 1–5 years (3, 0.4%) (Fig. 1). Temporally cases began in August (1 patient), numbered 70, 357, 148, and 9 in September, October, November, and December (Fig. 1).

**Table 1.** MAC-ELISA, GAC-ELISA and NS1Ag-ELISA result combinations in dengue patients (n=918).

**Test**	**Number of patients reactive (%)**
Anti-Dengue IgM capture ELISA	530 (76.9%)
Anti-Dengue IgG capture ELISA	433 (62.8%)
NS1 Antigen ELISA	415 (60.2%)
***Test Combinations reactive***	
**All three (IgM, IgG, NS1Ag) tests reactive**	217 (31.5%)
**Any two tests reactive**	
IgM+ve IgG+ve NS1Ag-ve	237 (34.4%)
IgM+ve IgG-ve NS1Ag+ve	63 (9.1%)
IgM-ve IgG+ve NS1Ag+ve	45 (6.5%)
**Any one test reactive**	
NS1Ag+ve only, IgM-ve, IgG-ve	90 (13.1%)
IgM+ve only, IgG-ve, NS1Ag-ve	13 (1.9%)
IgG+ve only, IgM-ve, NS1Ag-ve	24 (3.5%)

MAC-ELISA:anti-Dengue IgM capture ELISA, GAC-ELISA:anti-Dengue IgG capture ELISA, NS1Ag:NS1 Antigen.

Among these 689 patients, we prospectively observed 219 randomly selected patients (148 male, 71 female, 45 IgM+veIgG-veNS1Ag-ve, 69 IgM+veIgG-veNS1Ag+ve, 55 IgM+veIgG-veN-S1Ag-ve, 41 IgM+veIgG+veNS1Ag+ve and 9 IgM-veIgG+veNS1Ag+ve) (Table 1). Among these 219 patients, three (all male) patients died during hospital stay (Mortality 1.4%). As per WHO 1999 criteria, 4 patients (1.8%) were categorized as dengue shock syndrome, 7 (3.2%) as Dengue hemorrhagic fever, and 208 as dengue fever (95%) (Table 2).

**Table 2.** Characteristics of dengue patients (n=219) enrolled in observational study.

**Indicator**	**N**	**Mean Age ± 3SE**	**Mortality**
Participants	219 (100)	29.33 ± 2.5	3 (1.37) (+ 1 IUFD)
Gender			
Female	71 (32.4)	34.8 ± 4.8	0
Male	148 (67.6)	26.7 ± 2.6	3 (2.03)
**Classification of patients as per WHO 1997**			
Dengue Fever	162 (74.0)	29.7 ± 2.8	0
Dengue Hemorrhagic Fever	39 (17.8)	29.7 ± 5.5	2 (28.6)
Dengue Shock Syndrome	18 (8.2)	31.5 ± 10.5	1 (25.0)
**Classification of patients as per WHO 2009**			
Dengue fever without warning signs	39 (17.8)	27.9 ± 6.6	0
Dengue with warning signs	153 (69.9)	28.2 ± 2.8	0
Severe Dengue	27 (12.3)	32.1 ± 8.1	3 (11.1)
**Co-morbidities**			
Pregnancy	4 (1.8)	30 ± 14.7	One IUFD
Anaemia (Mild to moderate grade)	27 (12.3)	37.2 ± 8.6	1 (3.7)
Hypertension	13 (5.9)	47.1± 7.1	1 (7.7)
Diabetes mellitus	13 (5.9)	48.7 ± 5.5	1 (7.7)
Bronchial Asthma	5 (2.3)	44.8 ± 16.5	0
Hypothyroidism on supplemental L-Thyroxine	4 (1.8)	42.3 ± 7.8	0
Smokers	11 (5)	36.6 ± 9.9	0
Chronic Alcoholics	12 (5.5)	34.0 ± 9.9	1 (8.3)
Obesity	3 (1.4)	49.3 ± 3.6	0
Malnourishment	1 (0.5)	-	0

IUFD:Intra-uterine fetal death, WHO:World Health Organization.

### Epidemiological findings.

**Fig. 2:**
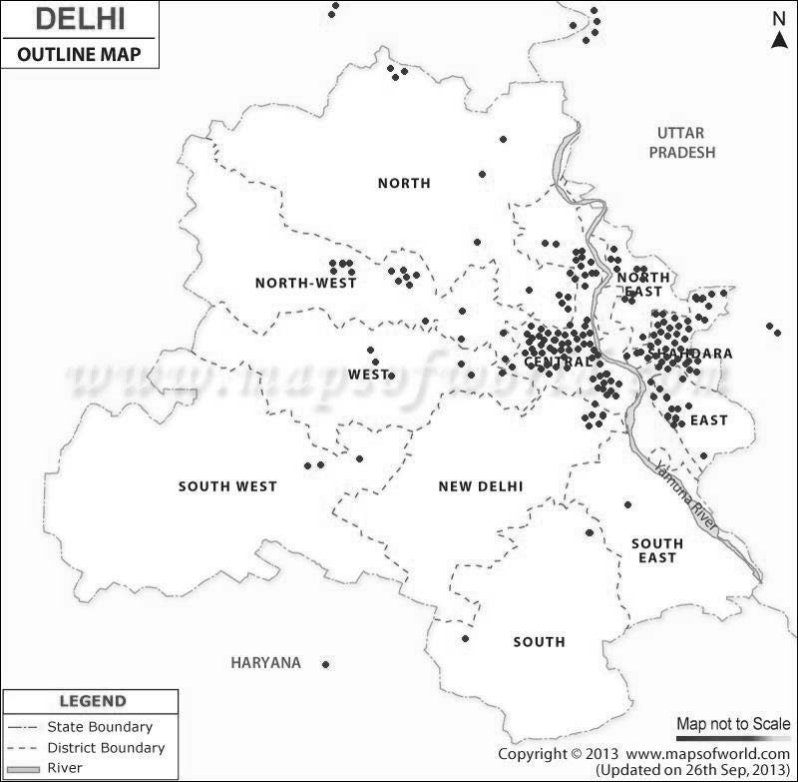
Residences of the 216 Dengue patients who were National Capital Region residents

Among the dengue patients, 149 were male and 70 females. 191 patients were of north-indian descent, 11 of northeast-indian descent, 11 south-indian, and 6 of east-indian descent. 216 patients were from National Capital Region (72 Trans-Yamuna River East Delhi, 3 from Dwarka, 2 from Gaziabad, 2 from Sonepat, Fig. 2) while 3 were travellers. Of those infected, 70 (32%) were students, 42 (19.2%) home-makers, 44 (20.1%) employed in various offices, 8 (3.8%) factory workers or mechanics, 2 (0.9%) retired, 2 (0.9%) unemployed, and one (0.5%) child below school going age. Mean monthly income of those gainfully employed was Rs 9872.00 (US$158.53); mean income loss in these patients due to illness was Rs. 2100.60 (US$33.73). Four (1.8%) patients gave history of past episode of dengue fever.

### Clinical and radiological findings.

**Fig. 3:**
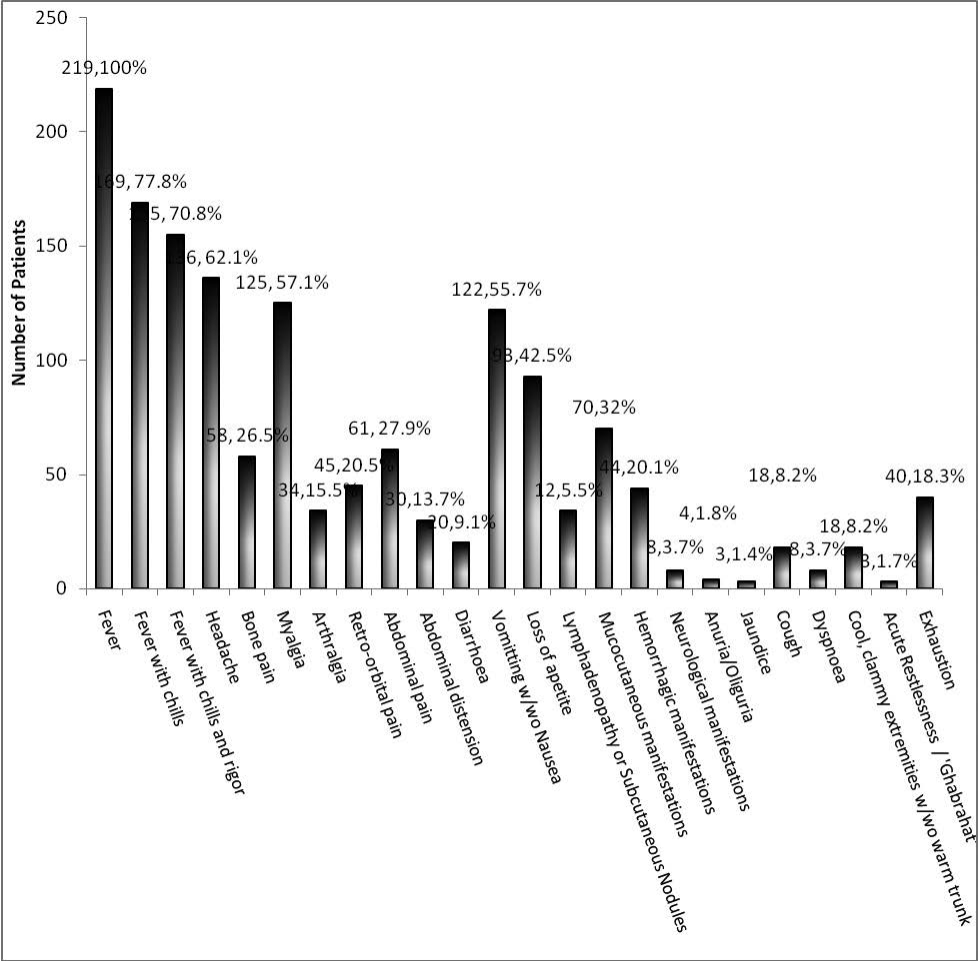
Clinical manifestations of dengue fever patients (n=219). w/wo : with / without, Ghabrahat:Hindi term meaning sense of unease/restlessness

The clinical findings among 219 dengue patients are detailed in Fig. 3. Fever duration ranged from 1 day to 28 days (mean 5.5 ± 0.4 days); maximum recorded temperature ranged from 101°C to 105°C (mean 102.5 ± 0.1°C) and saddle back fever was noted in 16 (7.3%) patients. The classical dengue triad was present in 55 patients (25.1%). A positive tourniquet test was noted in 11 patients of 155 patients tested. Hemorrhagic, neurologic and mucocutaneous manifestations were present in 44 (20.1%), 8 (3.7%) and 70 (32%) patients respectively and are detailed in Table 3. The secondary rash was maculopapular in 39 (73.6%) patients, petechial in 9 (15.3%), and macular in 5 (8.5%). The macular and maculopapular rashes were bilateral in 43 patients, started mostly on days 4 (10, 22.7%), 5 (11, 25%) and 6 (12, 27.3%), lasted a mean duration of 2.77 +/− 0.44 days, started distally at upper or lower limbs or both in 37 (82.2%) patients, on the neck in five, and centrally in two, was accompanied by itching on the rash in 8 (13.6%), and progressed to involve more central areas like upper arms, thighs, neck, chest, back and abdomen in 21 patients but remained localised to the initial site in 23 patients. Features of shock recorded include cool, clammy extremities with warm trunk (18, 8.2%), weak pulse (14, 6.4%), and blueness around mouth (2, 0.9%). Mean duration of onset of shock from the start of fever was 3.1 ± 1.2 days. Clinical examination revealed hepatomegaly in 18 (8.2%) patients and splenomegaly in one patient. Ultrasonographic assessment revealed enlarged liver (> 17 cm) in further 29 (13.2%) (total 47 (21.5%) patients with hepatomegaly), and an enlarged but clinically non-detectable splenomegaly in 41 (19.2%) patients (total 42 (19.2%) patients with splenomegaly). Of 42 patients with splenomegaly, 37 demonstrated only marginal enlargement of spleen (12.1–14.0 cm on largest dimension) while 5 patients demonstrated a little larger spleen (14.0–18.0cm). Ascitis, bilateral pleural effusion, and right sided pleural effusion was found in 53 (24.2%), 14 (6.4%), and 17 (7.7%) patients by a combination of clinical and radiological methods. Gall bladder wall oedema and thickening was noted in 45 (20.5%) patients.

**Table 3.** Hemorrhagic, neurologic and mucocutaneous manifestions noted in dengue patients.

**Hemorrhagic manifestation**	**Number of Patients (%)**
Easy bruising (especially at venipuncture site)	13 (5.9)
Petechiae	9 (4.1)
Fresh bleeding per Rectum	2 (0.9)
Epistaxis and Bleeding Gums	10 (4.6)
Bleeding Gums	5 (2.3)
Bleeding per-Vagina	2 (0.9)
Malena	8 (3.7)
Hematemesis	6 (2.7)
Hemoptysis	1 (0.5)
Hematuria	2 (0.9)
**Neurological manifestations**	
Impaired consciousness	7 (3.2)
Focal Neurological deficit	1 (0.5)
Seizures	1 (0.5)
**Mucocutaneous manifestations**	
Initial Exanthema (Rash)	20 (9.2)
Facial Flushing	2 (0.9)
Central flushing on chest & abdomen	6 (2.7)
Distal Flushing	13 (5.9)
Secondary Rash (Between Day1–10 of fever onset)	55 (25.1)
Conjunctival congestion	7 (3.2)
Oral blisters and ulcers	4 (1.8)
Itching	23 (10.5)
Itching over Rash	10 (4.6)

### Laboratory parameters.

**A] Hematological parameters:** Hematological parameters of the dengue patients are detailed in Table 4. Of note, 200 (91.1%), 111 (57.7%), 204 (93%), 93 (42.3%) patients respectively developed thrombocytopenia, leucopenia, reactive lymphocytes, and a rise in Absolute Lymphocyte count > 2000/ul during their hospital stay. Hematocrit was above the local cut-off in 212 (96.9%) patients, hemoconcentration > 20% and in 15–20% range was found in 43 (19.8%) and 28 (12.6%) patients respectively. A rise in eosinophils by 2.5% in differential was noted in 72 (32.8%) patients; a leukoerythroblastic blood film in 5 patients (2.3%) during some point in their hospital stay while left shift and smear cells were documented in 65 (29.7%) and 4 (1.9%) patients respectively. Mean ESR at 1 hour was 14.35±2.90 mm.
**B] Biochemical parameters:** Biochemical parameters of the dengue patients are detailed in Table 4. Mean ALT, AST, and alkaline phosphatase were 140.7±60.1 IU/dl, 237.8±115.0 IU/dl and 86.2±17.9 IU/dl respectively. Mean albumin: globulin ratio and ALT: AST ratio was 1.30 ± 0.07 and 0.70 ± 0.06 respectively.

**Table 4.** Hematological and biochemical parameters of 219 dengue patients.

**Hematological Parameters**	**Number of Patients (%)**		

	**At admission**	**Later during hospital stay**	**Overall**
Leukopenia (TLC < 4000/ul)	87 (39.8)	24 (10.8)	111 (50.6)
Leukocytosis (>11,000/ul)	9 (4.2)	19 (8.9)	28 (12.8)
ALC > 3500/ul	19 (8.5)	52 (23.9)	71 (32.4)
Rise of ALC > 2000/ul			93 (42.5)
Reactive Lymphocytes	126 (57.6)	78 (35.5)	204 (93.2)
Hemoconcentration ∼ 15–20%			28 (12.6)
Hemoconcentration > 20%			43 (19.8)
Hematocrit above cut-off	212 (96.9)		
Thrombocytopenia (<100,000/ul)	166 (75.7)	34 (15.4)	200 (91.3)
Eosinophilia (AEC> 500/ul)	2 (0.8)	35 (15.8)	37 (16.9)
≥2.5% ↑ in Eo% during hospitalization			72 (32.8)
Leukoerythroblastic blood film	3 (1.4)	2 (0.9)	5 (2.3)
Left shift	39 (14.6)	26 (9.7)	65 (29.7)
Smear cells	1 (0.5)	3 (1.4)	4 (1.9)
Deranged aPTT	15 (6.9)		
Deranged Prothrombin Time	7 (3.1)		
**Biochemical Parameters**	**Number of patients**		
Raised Alanine Transaminase	161 (73.5)		
Raised Aspartate Transaminase	184 (83.8)		
Hypoproteinemia (<6g/dl)	66 (30.2)		
Hypoproteinemia (<5g/dl)	12 (5.6)		
Hypoalbuminemia (<3g/dl)	35 (15.9)		
S. Creat.>1.2 mg/dl or s.BUN > 24 mg/dl	24 (11.1)		
Raised Alkaline phosphatase	14 (6.3)		
Hyperbilirubinemia^a^	13 (6)		

TLC: Total Leucocyte count, ALC:Absolute Lymphocyte count, AEC:Absolute Eosinophil count, S. Creat:Serum Creatinine, S. BUN:serum Blood Urea Nitrogen, Eo:Eosinophil, aPTT:activated partial Thromboplastin Time.

a: All 13 patients had predominant conjugated hyperbilirubinemia.

### Course of Illness.

167 (76.2%) patients’ required intravenous crystalloid fluid therapy, 2 patients in addition required intravenous colloid therapy; 69 (31.4%), 4 (1.5%), and 3 (1.1%) required platelet rich plasma transfusion, packed red blood cells, and fresh frozen plasma respectively. Out of 69 patients receiving platelet transfusion, 20 had active bleeding (29%), another 13 patients (18.8%) had platelet counts below 10 × 10^9^ / μl during their course of illness while 9 (13%) suffered shock and had an abnormal coagulogram. The rest 28 patients (40.6%) who received platelets had no appropriate indication as per NBVDCP guidelines (13). All 69 patients received 2 or more units (average 3.38 units received); no patient received single unit platelet transfusion. Close observation for development of danger signs and symptomatic treatment with anti-pyretics, analgesics and antiemetics was required for 46 (20.9%) patients. Renal replacement therapy in the form of hemodialysis was given to one (0.4%) patient. Twelve patients (4.5%) had to be treated in intensive care during hospital stay. Three patients (1.2%), (all male) died during hospital stay and there was one case of intra-uterine fetal death. Average duration of hospital stay was 6.3 ± 1.8 days. Two patients experienced hospital acquired blood stream infection (*Staphylococcus aureus* and *Klebsiella pneumoniae)* and three hospital acquired UTI (one each due to *Escherichia coli*, *Enterococcus faecalis*, and *Streptococcus bovis*) during their hospital stay. Four of the above recovered after change of venous lines, urinary catheters and appropriate antibiotic therapy. In the fifth patient, bilateral lower limb maculopapular rash got infected leading to lower limb cellulitis and pustular eruptions which yielded *Staphylococcus aureus* and *Streptococcus pyogenes* on culture. He further developed features of septicaemia (blood culture yielding *Staphylococcus aureus*). On Day-15 of hospital stay the patient deteriorated with myocarditis, arrhythmias and died on Day-18. The other two male patients who died (both on Day-5 of hospitalization) had features of dengue shock syndrome, with massive mucosal bleeds. Multi-organ involvement occurred in both of them (myocarditis, severe hepatitis and encephalopathy in one patient and severe hepatitis and encephalopathy in the other). All patients who recovered (216) were afebrile at the time of discharge and the mean platelet count at discharge was 129057 ± 14073 / ul. Mean expenditure per patient during the illness was calculated to be Rs.23492.00 (US$ 377.25).

## DISCUSSION

Outbreaks of dengue fever with consequent hospitalization of severe cases are becoming frequent since 1980s (12) especially in the south East Asian region. With no specific treatment available, prevention remains a cornerstone. Preventive measures are not effectively applied in developing countries due to lack of funding, trained manpower, community ignorance and non-participation (13). The morbidity and financial cost of this infection is tremendous on the afflicted subjects (14). In addition fatalities due to dengue fever are not uncommon as seen in our study (3 deaths, one IUFD). Majority of those hospitalised and two of those who died were young adults (20–40 years). The male predominance among those hospitalised may be due to rampant gender discrimination in India (15).

The seasonal distribution of dengue cases is well documented, with a peak incidence after the heaviest monsoon rainfall (12). The exact month of highest incidence may differ according to the timing of the rainfall. Mudpools, open water reservoirs and stagnant water in houses and roofs are breeding sites for *Aedes* (*Stegomyia*) *aegypti* mosquitoes (2, 12). Most affected patients were residents of NCR, mainly school and college going students and homemakers; however three travellers were also among those hospitalised. This finding highlights the serious situation of dengue in the capital; complicated by the lack of vaccines and chemoprophylaxis against the disease. Travellers and residents alike must strictly guard against mosquito bites through use of mosquito nets and repellents. Public health education regarding protection against mosquito bites is important but the growing resistance of mosquitoes to insecticides is a concerning factor (16).

Fever with chill and rigor, and aches were the commonest symptom. These apart vomiting and lack of appetite were very commonly noted. Many patients had troublesome persistent vomiting as their main complaint; abdominal pain was another significant trouble for some patients. Vomiting and abdominal pain have been previously documented to be particularly troublesome symptoms (17). Dengue triad was documented only in a quarter of the patients (25.1%). The tourniquet sign (11 out of 155 patients tested) was even more infrequently documented. The absence of the classical dengue triad or the tourniquet sign in many patients discourages the use of these for diagnosis or triage. In another two studies, one from Eastern India (17) and another from the north (4), positive tourniquet sign were found in 31% and 25.5% of dengue patients. Though hemorrhagic manifestations were noted in 44 patients, no individual site or lesion was responsible for majority of the cases; so it is important to be aware and on the look out of the myriad hemorrhagic presentations. Other studies have found similar wide distribution of hemorrhage patterns in dengue patients (4, 17–19). Of cutaneous manifestations, maculopapular or petiechial secondary skin rash was the most common manifestation, however initial flushing on the face or limbs or trunk was seen in minority (9.2%) of patients. Proportion of peteichial rash is similar to that seen patients from Lahore; however that with initial flushing is very low compared to same study (20). The macular or maculopapular rash occurred commonly between day-4 and day-6 of fever (74%), started distally (36, 72%) and progressed centrally or remained localized. Itching on the rash was reported only in minority (13.6%) unlike Afzar NA et al. who reported pruritus on 62% of rashes (20). In DSS patients’ onset of shock is usually around day 3 of fever (2, 12); in our study shock occurred most commonly between day 2 and day 4 of fever (61.1%), mean duration between onset of fever and shock being 3.1 ± 1.2 days. Hepatomegaly (47 patients, 20%), detected by combination of clinical and sonological examinations was a common feature. Though clinically imperceptible, splenomegaly was noticed in 41 (19.2%) patients on ultrasound examination. Majority of patients with splenomegaly had marginal enlargement while five demonstrated a moderately large spleen (14.0–18.0cm). Similarly, another study noted 27/70 patients with clinical hepatomegaly and 9/70 with only sonological evidence while the same with splenomegaly was 3/70 and 15/70 patients respectively (21). Other markers of plasma leakage (2, 12) were also detected better by radiological methods including ascitis and pleural effusion in 24.2% and 14.1% patients respectively, as noted in other studies (17, 21). Further we noted that right sided pleural effusion was earlier to appear and pleural effusion was often uni-lateral. Many such minimal effusions were detected more frequently by USG than the conventional CXR. Thickened and oedematous gall bladder wall changes are characteristic of the plasma leakage syndrome in dengue (22) and may be marker of severe dengue (22).

Significant thrombocytopenia, marked leucopenia (50.6%) and lymphocytosis (32.8%) with presence of many reactive lymphocytes in the peripheral smear (93%) are commonly noted features in dengue fever. In our laboratory we often utilize these parameters to suspect a dengue case during outbreak periods. Together with significant transamnitis (83.4% patients) and hemoconcentration (19.8%), these findings are a valuable clue to the laboratory regarding the diagnosis. Interestingly, in addition a rise in eosinophil count is often documented later in the course of dengue as seen in 32.8% of our patients. The cut-off levels of Hb% as a marker for hemoconcentration devised previously (4, 8) seems to need revision as were many as 96.9% dengue patients were above cut-off at admission. In contrast, however, hemoconcentration in excess of 20% and 15% was documented in only 19.8% and 32.4% patients only. The cut-off levels need to indicate only the at-risk patients (those who will go on to develop severe dengue or DHF) (8) and not all cases of dengue. Leukopenia occurred in more than half of dengue patients; in contrast Shukla V et al. found leucopenia in only 10% of cases (21). As noted in our study, transamnitis with ALT and AST greater than double the upper normal range is documented in majority of patients (16, 21, 23). The AST: ALT ratio 1.42 which is similar to that of 1.8 and 2.0 seen in other studies (21, 23). More severe liver dysfunction as indicated by hyperbilirubinemia, deranged coagulation profile, raised alkaline phosphatise and significant hypoproteinemia (and hypoalbuminemia) are found in only a minority of cases and indicates severe disease (24). Reversible renal dysfunction was also found in 24 (11.1%) patients. A leukoerythroblastic blood film and smear cells are found in even fewer patients of dengue hemorrhagic fever. Leukoerythroblastic blood film probably represents a bad prognostic factor in dengue patients as 3 out of the 5 patients died of the disease.

A combination of dengue antigen and antibody detection by ELISA tests was used in our study to confirm the diagnosis. However, MAC-ELISA and GAC-ELISA can show serological cross-reactivity among flavivirus infections like Murray Valley fever, Japanese Encephalitis, St. Louis Encephalitis, Yellow fever and West Nile (2, 25). Rheumatoid factor, malaria, Chikungunya, Lyme disease, Scrub Typhus, Hanta virus infection and leptospirosis are additional scenarios producing false positive MAC-ELISAs (2, 25, 26). A persisting IgM against Dengue virus originating from a previous infection will result in false diagnosis (27, 28). IgM anti-Dengue usually persists for 2–6 months (28, 29) with a median time period of 179 days for primary and 139 days for secondary infections (29). Anti-Dengue IgG antibodies persists for an even longer time even up to years (2, 30). False positive anti-Dengue virus IgG detection has been documented in bacteremia, leptospirosis, Q fever, and other viral infections like Chikungunya, Tick-borne encephalitis, Varicella, Cytomegalovirus, and Epstein-Barr infections (28, 31). False negative GAC-ELISA can occur in primary dengue infections where these antibodies are slow to appear (32). Testing GAC-ELISA on paired samples is thus more worthwhile (2, 27). NS1Ag is often only transiently present in secondary dengue infections and may be masked by antigen-antibody complexes. False positive NS1Ag tests have been detected in acute Zika virus infection, Cytomegalovirus infection and in patients with haematological malignancies (33–35). In light of such discrepancies, clinical diagnosis in our hospital depends on a combination of antigen and antibody detection rather than a single assay.

Close observation and symptomatic management is all that is required in most cases of simple dengue (2, 12). Anti-pyretic were nearly universally prescribed for these patients while anti-emetics were required in considerable number of patients. Careful intravenous fluid replacement remains the cornerstone for managing severe cases of dengue (2, 12, 17). 167 of our patients received intravenous crystalloids as therapy; fluid overload was monitored for in these cases. Fluid overload can lead to serious consequences (17). Two of our patients required intravenous colloid solutions while four patients were treated with fresh frozen plasma for refractory shock. Most authors report no relation between platelet counts and clinical bleeds, however the duration of shock has important implications regarding severe bleeding (11). Thus appropriate monitoring of shock and sequential hematocrit monitoring may have been more appropriate (2, 11, 12). Active bleeding may be a more appropriate indication platelet transfusion rather than low platelet count. Only 29% of dengue related platelet transfusions in our study population had active bleeding while 40.6% patients had no appropriate indications as per NBVDCP guidelines (11). Other hospitals in Delhi also report high usage of platelet transfusions, often inappropriately (3, 5). WHO as well as the Indian NBVDCP authorities recommend packed cell transfusion in only those cases who show 10% or more loss of blood volume and in those with refractory shock with declining hematocrit (11, 12). In addition to platelets, four patients required such packed red cell transfusion while three patients were given fresh frozen plasma in view of coagulopathy.

Mortality in our study was 1.2%; additionally there was a case of intra-uterine fetal death. All three dead patients were male, bread-winners of their family and belonged to 20–55 years working age group. One patient died due to refractory shock along with myocarditis and severe hepatic involvement; the second due to refractory shock with severe blood loss and hepatic involvement superadded on chronic renal failure and diabetes mellitus; and the third due to myocarditis, cellulitis and staphyloccal sepsis. Severe hepatic involvement with transaminases greater than 1000 IU/dl and myocarditis have previously also been associated with poor prognosis. The morbidity due to dengue is no less, mean duration of hospital stay was more than 6 days in our study, mean expenditure per patient due to the illness was greater than US$377; and further nosocomial infections complicated the course in as many as 5 patients. Shepard DS et al. in their pan-India study conducted from 2006–2012 who noted an average expenditure of $235.20 in a hospitalised patient in private set-up (14). The higher expenditure in our study may reflect inflationary changes and the fact that Delhi is one of the costliest cities in India.

A major limitation in our study is the dependence of dengue diagnosis on serological and antigen detection assays only. Due to resource limitations, viral RNA detection and virus culture was not possible. Further, serotype / genotype identification could not be done during the study.
